# A nomogram for predicting the risk of persistent coronary artery aneurysms in children with Kawasaki disease: a retrospective study

**DOI:** 10.3389/fcvm.2026.1741197

**Published:** 2026-01-22

**Authors:** Shang Lifeng, Su Danyan, Qin Suyuan, Chen Cheng, Qiao Xiaoyu, Sun Lu, Wang Zhouping, Pang Yusheng

**Affiliations:** 1 The First Affiliated Hospital of Guangxi Medical University, Nanning, Guangxi, China; 2 Heart Center and Institute of Pediatrics, Guangdong Provincial Clinical Research Center for Child Health, Guangzhou Women and Children’s Medical Center, Guangzhou Medical University, Guangzhou, China

**Keywords:** coronary artery aneurysm, Kawasaki disease, nomogram, predictive model, total bile acid

## Abstract

**Objective:**

To develop and validate a nomogram for the individualized prediction of persistent coronary artery aneurysms (CAAs) in children with Kawasaki disease (KD) who have developed CAAs in the acute phase.

**Methods:**

This retrospective cohort study enrolled children diagnosed with KD and complicated by CAA between September 2015 and December 2023. The primary outcome was defined as the persistence of CAA 90 days after disease onset. Predictor selection was performed using 1,000 bootstrap resamples combined with LASSO regression for stability. A predictive model was constructed using multivariate logistic regression. The model's discrimination, calibration, and clinical utility were assessed by the area under the receiver operating characteristic curve (AUC), calibration curves, and decision curve analysis (DCA).

**Results:**

A total of 135 children were included, of whom 80 (59.3%) had persistent CAAs. Stability selection identified the maximum coronary artery *Z*-score (ZM), age < 12 months (Age1), and total bile acid (TBA) as key predictors. The parsimonious model (Model B) built on these predictors demonstrated excellent performance, with an optimism-corrected AUC of 0.933 (95% CI: 0.905–0.960). It was well-calibrated, and DCA showed a positive net benefit across a wide threshold probability range of 5%–100%.

**Conclusion:**

This study successfully developed a nomogram based on ZM, Age1, and TBA. This tool can effectively identify KD children at risk of persistent CAAs, providing an intuitive and quantitative decision-making aid for precise risk stratification and optimized long-term management in this high-risk population.

## 1 Introduction

Kawasaki disease (KD) is one of the most common acute systemic vasculitides in childhood, predominantly affecting children under 5 years of age (1). The core pathological feature is systemic vascular inflammation, with the coronary arteries being the most susceptible to involvement (2). Persistent inflammatory responses disrupt the structural integrity of the vascular wall and are a key driver of coronary artery aneurysm (CAA) formation (3). Although intravenous immunoglobulin (IVIG) has significantly reduced the overall incidence of coronary artery lesions, epidemiological data indicate that approximately 3%–5% of children with acute KD still develop CAAs (4). The clinical course of CAAs is highly heterogeneous. While many aneurysms regress over time, especially in children treated early with IVIG, a significant proportion persists, forming the pathological basis for thrombosis and coronary stenosis (5). Studies show that among children with medium-sized CAAs, about 10% have persistent aneurysms, of which approximately 20% may progress to coronary stenosis due to thrombosis (6). These persistent lesions significantly increase the long-term risk of cardiovascular events, including myocardial ischemia, arrhythmias, and even sudden cardiac death (7).

Given their potential long-term cardiovascular harm, guidelines from the American Heart Association (AHA) classify KD with persistent CAAs in the highest cardiovascular risk category (8). This classification underscores the necessity for early identification and intensive management of this group. Although several clinical indicators associated with CAA persistence have been identified (5, 9, 10), there remains a lack of effective clinical tools that integrate these key predictors to address the crucial question of “whether an existing CAA will persist” and provide both intuitive and individualized risk quantification (11).

In developing such predictive models, the robustness of variable selection is crucial for ensuring model reproducibility. Traditional variable selection methods are susceptible to the randomness of a single dataset, potentially leading to overfitting or the inclusion of false-positive predictors. Therefore, this study innovatively employs bootstrap-LASSO stability selection to rigorously screen variables, aiming to identify the most reproducible core predictors from numerous candidates, thereby fundamentally enhancing the model's robustness and generalizability.

Nomograms have recently gained prominence as intuitive clinical prediction models. By transforming complex regression models into graphical scoring systems, they allow clinicians to quickly obtain individualized risk assessments. This study aims to construct a nomogram model based on stable variable selection to predict the risk of persistent CAAs in children with KD, providing a quantitative tool for clinical risk stratification and individualized management decisions.

## 2 Methods

### 2.1 Study population

This retrospective cohort study enrolled children diagnosed with Kawasaki disease and complicated by coronary artery aneurysm (CAA) at the Guangzhou Women and Children's Medical Center between September 2015 and December 2023. Inclusion criteria were: diagnosis of CAA according to the American Heart Association (AHA) 2017 KD guidelines (coronary artery *Z*-score ≥2.5). Exclusion criteria were: 1) congenital heart disease or rheumatic disease; 2) missing >20% of key clinical, laboratory, or echocardiographic follow-up data; 3) IVIG or corticosteroid treatment prior to admission; 4) recurrent KD; 5) coronary dilation secondary to cardiomyopathy or congenital coronary anomalies. All children received standard IVIG treatment (a single infusion of 2 g/kg) after onset, along with antiplatelet/anti-inflammatory therapy per guidelines. Following discharge, patients were routinely monitored with echocardiography at approximately 1 week, 1 month, 2 months, and 3 months. For the purpose of this predictive model study, predictor variables were defined using data from the acute phase (pre-IVIG), and the primary outcome was determined solely by the echocardiogram obtained at the 3-month (90 ± 7 days) time point.

### 2.2 Outcome definition

Persistent coronary injury was defined according to the AHA 2017 guidelines as the presence of coronary injury on echocardiogram performed ≥90 days after IVIG treatment. Coronary artery dimensions were adjusted for body surface area and expressed as *Z*-scores, calculated using the regression equation established by Dallaire et al. Coronary injury was graded by *Z*-score: <2.0 = normal, 2.0–2.4 = dilation, 2.5–4.9 = small aneurysm, 5.0–9.9 = medium aneurysm, ≥10.0 = giant aneurysm. The primary outcome was defined as the persistence of CAA 90 days (±7 days) after disease onset, determined by a *Z*-score ≥2.5 in any coronary segment at follow-up. For patients with IVIG resistance, the standard rescue therapy at our center was a second dose of IVIG combined with corticosteroids.

### 2.3 Data collection

The maximum coronary artery *Z*-score (body surface area-adjusted) was independently assessed by two cardiac sonographers blinded to group assignment. Predictor variables included: Demographics: age, sex, height, weight. Clinical features: KD type (complete/incomplete), IVIG resistance (recurrent fever >36 h after initial IVIG completion), delayed IVIG treatment (>10 days after fever onset), specifically whether the child was an infant [Age_Infant: age <12 months (yes) vs. age ≥12 months (no)]. Coronary parameters: maximum coronary artery *Z*-score in the acute phase (before IVIG and within 30 days after IVIG). Laboratory indicators: (all collected before IVIG administration): inflammatory markers (CRP, ESR, PCT), complete blood count (WBC, NEU, Hb, PLT), liver and renal function (ALT, AST, Cr), coagulation profile (PT, APTT, Fib), myocardial injury markers (CK-MB). Data were extracted from the structured electronic medical record system and entered independently by two researchers with cross-checking. To ensure methodological consistency across the entire cohort, coronary *Z*-scores for all patients, including those enrolled from 2015 to 2017, were (re)calculated using the uniform equation specified in the Outcome Definition section (i.e., the Dallaire et al. formula). This process guaranteed that the same diagnostic standard was applied to every participant. All echocardiographic examinations were performed with the child quiet or sedated, in the standard left lateral decubitus position. Examinations followed a systematic assessment protocol, sequentially scanning the parasternal long-axis, short-axis, and apical views. Coronary artery internal diameters were measured at end-diastole at standard proximal locations of the following coronary segments: left main coronary artery (LMCA), left anterior descending artery (LAD), left circumflex artery (LCx), and right coronary artery (RCA). The key anatomical assessment metric for this study was the maximum coronary artery *Z*-score (ZM), defined as the highest body surface area-adjusted *Z*-score measured in any of the above coronary segments during the acute phase.

### 2.4 Statistical analysis

Continuous variables were tested for normality using the Shapiro–Wilk test. Normally distributed data are presented as mean ± standard deviation and compared using the unpaired Student's *t*-test; non-normally distributed data are presented as median (interquartile range) and compared using the Mann–Whitney *U*-test. Categorical variables are presented as number (percentage) and compared using the χ^2^ test or Fisher's exact test. Internal validation was performed using 1,000 bootstrap resamples (12). Predictor selection employed LASSO regression (13) combined with bootstrap stability selection. A multivariate logistic regression model was built using the selected predictors, and odds ratios (ORs) with 95% confidence intervals (CIs) were calculated. The model's discrimination, calibration, and clinical utility were evaluated using the area under the receiver operating characteristic (ROC) curve, calibration plots, and decision curve analysis (DCA) It should be noted that the predictor data were largely complete. Specifically, white blood cell count (WBC) was missing in 5 cases (3.7%), and NT-proBNP was missing in 7 cases (5.2%). Data for total bile acids (TBA) and all other variables were fully available, with all missing proportions well below the 20% exclusion criterion. These limited missing data were handled using multiple imputation by chained equations (14). All analyses were performed using R software (version 4.2.2). A two-sided *P*-value < 0.05 was considered statistically significant.

### 2.5 Ethics statement

The retrospective data used in this study were entirely sourced from the Guangzhou Women and Children's Medical Center. The study protocol was reviewed by the Ethics Review Committee of the Guangzhou Women and Children's Medical Center, Guangzhou Medical University. As this is a retrospective study utilizing anonymized data, the committee granted an exemption from ethical review (Approval No: 2025-389B00) and waived the requirement for informed consent.

## 3 Results

### 3.1 Baseline characteristics of the study population

This study included 135 children with Kawasaki disease (KD) and coronary artery aneurysms (CAA), of whom 80 (59.3%) had persistent CAAs at the 3-month follow-up after intravenous immunoglobulin (IVIG) treatment. Compared to the regressed CAA group, the persistent CAA group demonstrated significantly higher acute-phase maximum coronary *Z*-scores (ZM) [4.62 (3.72; 5.68) vs. 2.73 (2.61; 3.09)], higher white blood cell counts [19.13 (17.74; 22.76) × 10⁹/L vs. 17.42 (15.33; 19.68) × 10⁹/L], a greater proportion of children aged <12 months (47.50% vs. 18.18%), and a higher rate of delayed IVIG treatment (40% vs. 7.27%). All these between-group differences were statistically significant (*P* < 0.001). Additionally, significant differences (*P* < 0.05) were observed in the proportion with C-reactive protein (CRP) >100 mg/L, height, weight, original CRP values, age, and absolute neutrophil count. The detailed baseline characteristics are presented in Table 1.

**Table 1:** Characteristics of the study population.

Variables	Regressed CAA (*N* = 55)	Persistent CAA (*N* = 80)	*P*
Gender:			
Female	14 (25.45%)	17 (21.25%)	0.717
Male	41 (74.55%)	63 (78.75%)	
Resistan:			
No	50 (90.91%)	68 (85.00%)	0.452
Yes	5 (9.09%)	12 (15.00%)	
Age (month)	24.00 [15.00; 34.50]	14.50 [6.00; 28.00]	0.001***
Kg	11.50 [9.30; 13.75]	10.00 [7.95; 12.93]	0.012*
M	0.85 [0.78; 0.94]	0.79 [0.69; 0.90]	0.009**
Incomplete:			
No	38 (69.09%)	45 (56.25%)	0.185
Yes	17 (30.91%)	35 (43.75%)	
White blood cell (WBC ×10^9^/L)	17.42 [15.33; 19.68]	19.13 [17.74; 22.76]	<0.001***
Red blood cell (RBC ×10^12^/L)	4.18 [3.84; 4.57]	4.00 [3.72; 4.38]	0.117
Hemoglobin (HB g/L)	102.93 (12.83)	99.35 (11.17)	0.096
Platelet (PLT ×10^9^/L)	395.00 [300.00; 513.50]	437.50 [338.50; 646.75]	0.057
Neutrophils (NEU ×10^9^/L)	11.72 [9.23; 14.50]	13.73 [11.00; 15.82]	0.013*
Neutrophils% (NEU %)	67.84 (11.99)	68.67 (13.50)	0.708
CRP (mg/L)	102.65 [68.62; 119.55]	113.02 [100.57; 139.10]	0.018*
PCT (ng/ml)	0.56 [0.30; 2.14]	1.59 [0.40; 3.18]	0.111
ESR (mm/h)	52.00 [37.50; 80.50]	48.00 [29.75; 74.25]	0.339
NT_proBNP (pg/ml)	951.00 [310.26; 1,743.49]	418.57 [198.67; 2,268.75]	0.384
K^+^ (mmol/L)	3.79 (0.46)	3.84 (0.60)	0.556
Na^+^ (mmol/L)	135.90 [135.20; 137.95]	136.60 [134.78; 138.10]	0.761
Cl^−^ (mmol/L)	98.90 [97.95; 101.95]	98.70 [96.77; 100.82]	0.111
Ca^2+^ (mmol/L)	2.27 [2.14; 2.35]	2.27 [2.16; 2.37]	0.574
Total Bilirubin (TBil umol/L)	5.00 [3.30; 6.90]	4.75 [2.90; 8.05]	0.97
Direct bilirubin (umol/L)	2.00 [1.25; 3.30]	2.10 [1.28; 4.93]	0.565
Indirect bilirubin (umol/L)	2.70 [1.85; 3.50]	2.45 [1.50; 3.92]	0.71
Total Protein (TP g/L)	63.57 (7.25)	65.11 (7.84)	0.242
Albumin (Alb g/L)	34.90 [31.65;38.05]	34.70 [31.23; 36.78]	0.4
Globulin (Glob g/L)	27.00 [24.45;31.45]	29.70 [25.78; 35.38]	0.059
Alb/Glob	1.26 (0.30)	1.15 (0.36)	0.077
γ-Glutamyl transpeptidase (γ-GT GGT U/L)	55.00 [27.00; 91.00]	65.50 [23.75; 130.00]	0.452
Total Bile Acids (TBA umol/L)	6.70 [3.95; 12.30]	9.00 [4.68; 19.90]	0.056
Aspartate aminotransferase (AST U/I)	28.00 [23.00; 35.50]	26.00 [21.00; 38.00]	0.458
Alanine aminotransferase (ALT U/I)	33.00 [20.00; 74.00]	30.00 [15.75; 64.25]	0.286
Alkaline phosphatase (ALP U/L)	194.00 [144.50; 215.50]	174.00 [125.75; 230.25]	0.327
Lipase (U/L)	34.00 [27.50; 47.50]	38.00 [30.00; 51.25]	0.279
Urea nitrogen (Urea mmol/L)	3.00 [2.33; 3.59]	3.12 [2.60; 3.62]	0.64
Creatinine (Cr umol/L)	22.00 [19.00; 25.50]	19.50 [15.75; 25.25]	0.056
Uric Acid (UA umol/L)	182.00 [149.50; 239.50]	193.00 [155.00; 231.00]	0.542
Lactate (mmol/L)	2.08 [1.64; 2.50]	2.02 [1.70; 2.78]	0.522
CystatinC (umol/L)	0.80 [0.73; 0.90]	0.86 [0.75; 0.97]	0.106
Creatine kinase (CK, U/L)	33.00 [21.00; 61.00]	28.50 [17.75; 43.75]	0.174
Creatine kinase-myocardial band (CKMB, U/L)	13.00 [9.50; 17.00]	13.50 [11.00; 21.00]	0.31
CK/CKMB	0.42 [0.26; 0.58]	0.52 [0.29; 0.78]	0.056
Lactate dehydrogenase (LDH U/L)	262.00 [233.50; 299.00]	254.50 [221.00; 281.50]	0.238
α_HBDH (U/L)	217.00 [194.00; 244.00]	208.00 [187.00; 232.25]	0.271
Thrombin activity (%)	89.00 [84.50; 101.00]	93.00 [82.00; 105.00]	0.356
Activated partial thromboplastin time (APTT s)	39.50 [36.50; 43.25]	40.40 [35.68; 44.95]	0.667
Prothrombin time (PT s)	13.70 [13.15; 14.30]	13.80 [13.07; 14.43]	0.98
Fibrinogen (Fib g/L)	6.36 (1.16)	6.02 (1.28)	0.11
Thrombin time (TT s)	17.01 (1.68)	16.47 (1.58)	0.062
INR	1.06 [0.99; 1.10]	1.06 [1.00; 1.13]	0.781
Age1 (age <12 month):			
No	45 (81.82%)	42 (52.50%)	0.001***
Yes	10 (18.18%)	38 (47.50%)	
CRP1:			
No	25 (45.45%)	19 (23.75%)	0.014*
Yes	30 (54.55%)	61 (76.25%)	
IVIG_delay:			
No	51 (92.73%)	48 (60.00%)	<0.001***
Yes	4 (7.27%)	32 (40.00%)	
Maximum *Z*-Score (ZM)	2.73 [2.61; 3.09]	4.62 [3.72; 5.68]	<0.001***

* *P* < 0.05.

** *P* < 0.01.

*** *P* < 0.001.

### 3.2 Stable predictors identified by bootstrap and LASSO regression

In clinical prediction model development, the robustness of variable selection is crucial for ensuring model reproducibility. To avoid overfitting or the inclusion of false-positive predictors due to randomness in a single dataset, we employed the rigorous bootstrap-LASSO stability selection method. This approach involved 1,000 bootstrap resamples to simulate data variation. LASSO regression (with 10-fold cross-validation, using *λ*min as the optimal penalty parameter) was run on each resample. The selection frequency of each variable across all resamples served as a quantitative measure of its predictive stability. The results showed that the maximum coronary *Z*-score (ZM) was selected in all 1,000 resamples (selection frequency 100%). Age <12 months (Age1) and total bile acid (TBA) demonstrated selection frequencies of 97.9% (979/1,000) and 90.0% (900/1,000), respectively. Variables with selection frequencies ≥80% also included lipase (87.2%, 872/1,000) and delayed IVIG treatment (83.0%, 830/1,000). The specific selection frequencies for all variables are detailed in Table 2.

**Table 2:** Stable predictors identified by bootstrap and LASSO regression.

Variables assessed	Frequency	Proportion
ZM	1,000	100.00%
Age1	979	97.90%
TBA	900	90.00%
Lipase	872	87.20%
IVIG_delay	830	83.00%
FIB	797	79.70%
PCT	740	74.00%
CK/CKMB	648	64.80%
GGT	563	56.30%
AST	549	54.90%
Cr	534	53.40%
ALT	531	53.10%
Glob	530	53.00%
WBC	505	50.50%
CystatinC	475	47.50%
ESR	473	47.30%
Cl-	471	47.10%
CRP1	445	44.50%
INR	432	43.20%
K+	425	42.50%
Lactate	393	39.30%
NEU	380	38.00%
RBC	379	37.90%
Gender	376	37.60%
UA	369	36.90%
TP	340	34.00%
Resistan	313	31.30%
Ca2+	312	31.20%
TT	310	31.00%
incomplete	295	29.50%
Thrombin activity	285	28.50%
Na+	261	26.10%
Hb	252	25.20%
NT_proBNP	250	25.00%
Urea	249	24.90%
PLT	245	24.50%
LDH	242	24.20%
Alb	219	21.90%
CKMB	202	20.20%
CRP	197	19.70%
α_HBDH	188	18.80%
ALP	158	15.80%
IBil	157	15.70%
Age	148	14.80%
m	118	11.80%
Alb_Glob	107	10.70%
NEU%	102	10.20%
APTT	81	8.10%
Kg	78	7.80%
DBil	34	3.40%
PT	33	3.30%
CK	32	3.20%
TBil	7	0.70%

### 3.3 Model development, performance, and statistical comparison

Using bootstrap-LASSO stability selection (1,000 resamples) and pre-defined variable selection frequency thresholds (100%, 90%, 80%), we constructed three candidate models (Model A, B, C). Internal validation yielded an optimism estimate of 0.031 (95% CI: 0.010–0.061). Model performance was evaluated using optimism-corrected predicted probabilities, while statistical comparisons between models were based on the original predicted probabilities or classification results (detailed in Tables 3, 4).

**Table 3:** Comprehensive performance and statistical comparison of prediction models.

Metric	Model A	Model B	Model C
Number of variables	1	3	5
Original AUC (95% CI)	0.924 (0.881–0.967)	0.964 (0.936–0.991)	0.969 (0.942–0.996)
Corrected AUC (95% CI)	0.893 (0.850–0.935)	0.933 (0.905–0.960)	0.938 (0.911–0.965)
Brier score	0.109	0.076	0.063
Calibration (H-L test)	χ^2^ = 3.692, *p* = 0.884	χ^2^ = 11.813, *p* = 0.160	χ^2^ = 10.500, *p* = 0.232
DCA useful range	0.10–1.00	0.05–1.00	0.04–1.00
DCA range length	0.89	0.94	0.95

**Table 4:** Statistical comparison table (using model B as reference).

Comparison Item	Model A vs. Model B	Model B vs. Model C
DeLong's test (*Z*, *p*)	*Z* = −2.415, *p* = 0.016	*Z* = −0.861, *p* = 0.389
McNemar's test (χ^2^, *p*)	χ^2^ = 0.500, *p* = 0.479	χ^2^ = 0.364, *p* = 0.546

#### 3.3.1 Discrimination

**Figure 1:**
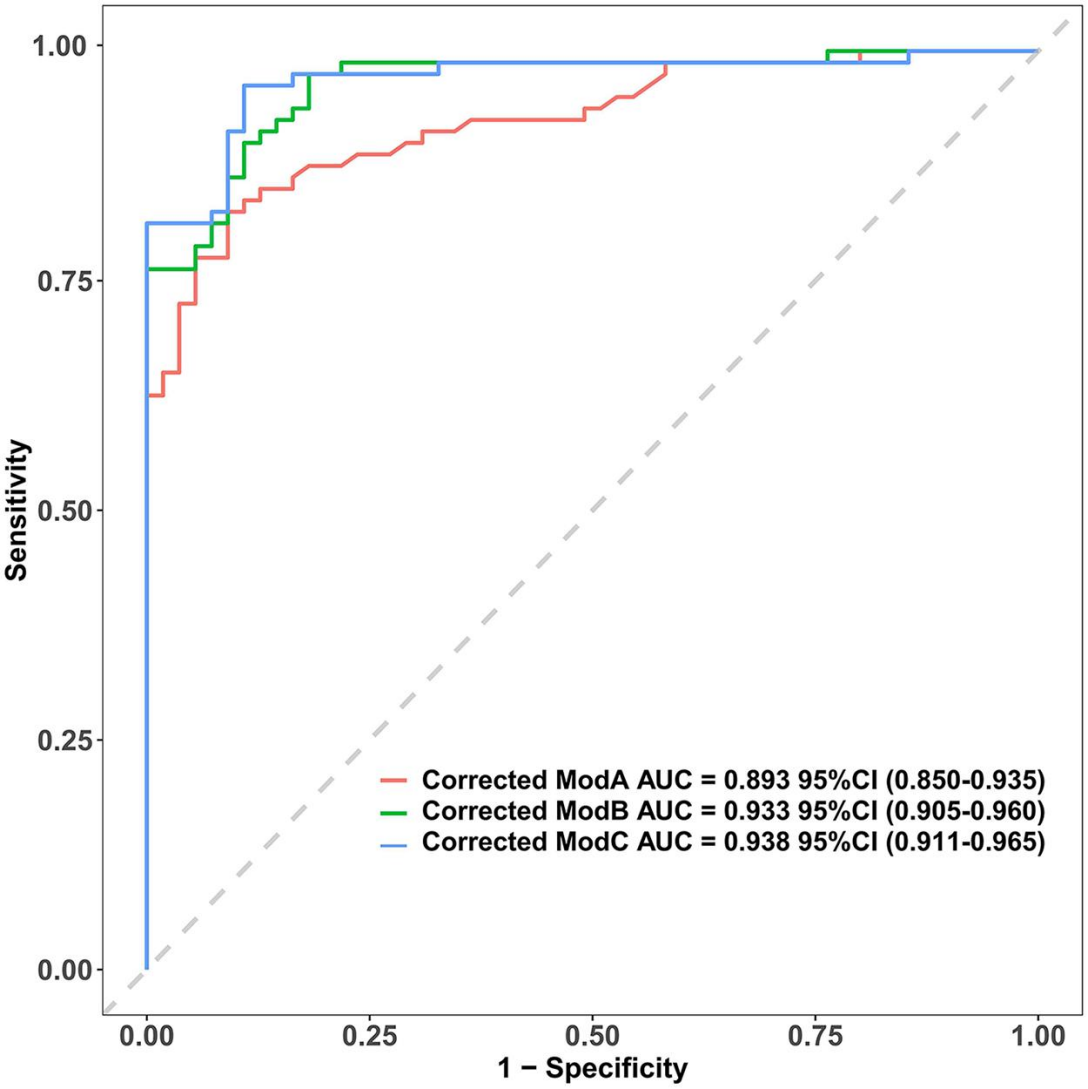
Comparison of ROC Curves for the Three Adjusted Prediction Models. The corrected area under the curve (AUC) with 95% confidence intervals for Model A, Model B, and Model C are 0.893 (0.850–0.935), 0.933 (0.905–0.960), and 0.938 (0.911–0.965), respectively. The diagonal grey line represents a reference for a model with no discriminative power (AUC = 0.5). AUC, area under the curve.

As shown in Table 3, the corrected AUCs for Model B and C were significantly higher than that for Model A (DeLong's test, *p* < 0.05). The ROC curves for Model B and C nearly completely overlapped (Figure 1), consistent with the statistical test result showing no significant difference in their discrimination (*Z* = −0.861, *p* = 0.389). Thus, both Model B and C exhibited excellent and comparable discriminatory performance, which was significantly superior to Model A, which contained only ZM.

#### 3.3.2 Calibration

**Figure 2:**
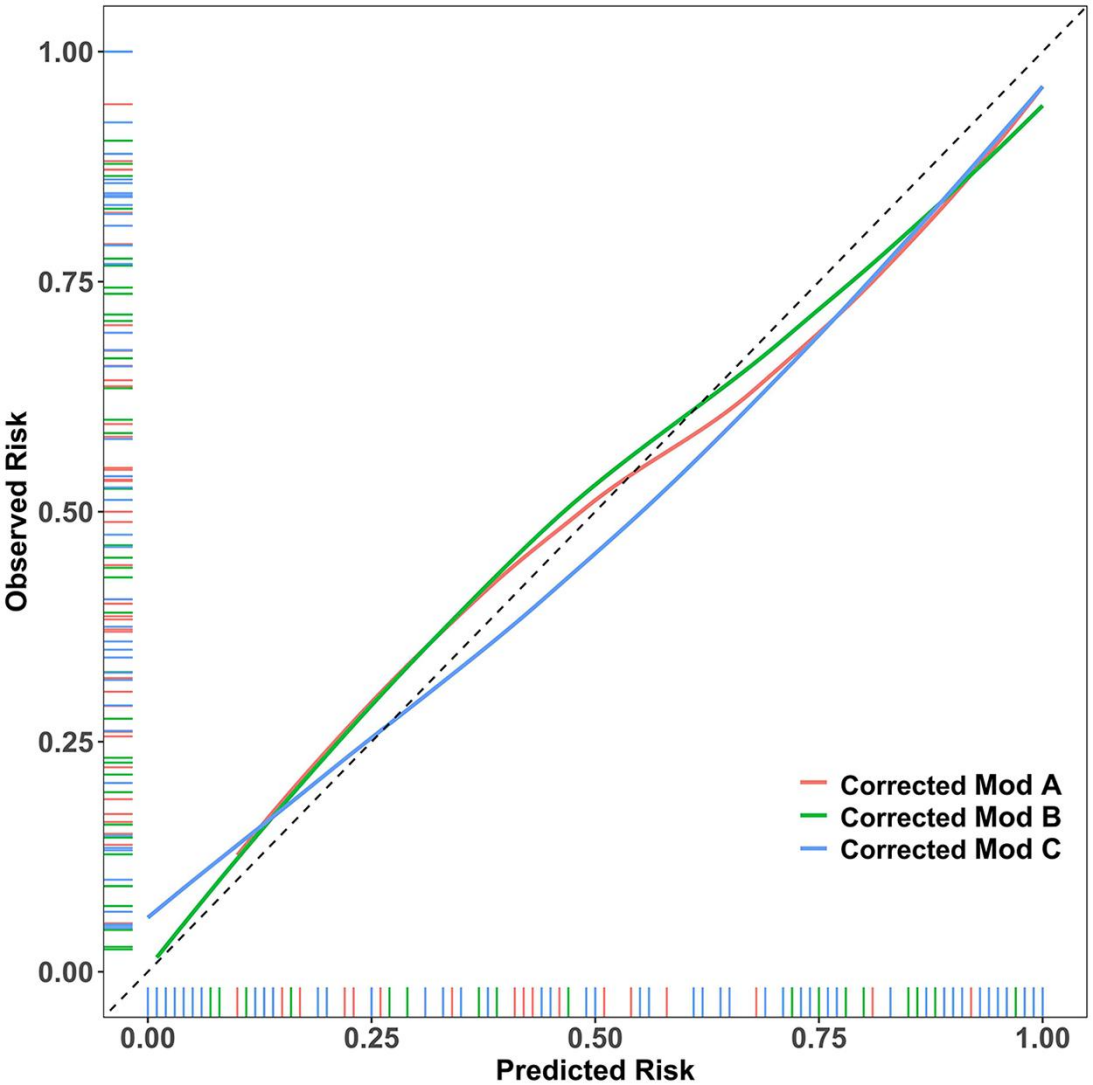
Assessing Model Calibration Using Optimism-Corrected Probabilities. Showing the calibration curves generated from bootstrap optimism-corrected probabilities, this figure reveals that all three models closely follow the ideal reference line, demonstrating minimal calibration error.

The Hosmer-Lemeshow test *p*-values were greater than 0.05 for all models, indicating no significant calibration bias. As shown in the calibration curves (Figure 2), which were plotted using optimism-corrected probabilities, the predicted probability curves for all three models closely followed the ideal reference line, visually confirming the accuracy of the predicted probabilities. This demonstrates that both the parsimonious Model B and the more complex Model C achieved predicted risk probabilities that were highly consistent with the actual observed probabilities, confirming the reliability of the model predictions.

#### 3.3.3 Clinical utility: decision curve analysis

**Figure 3:**
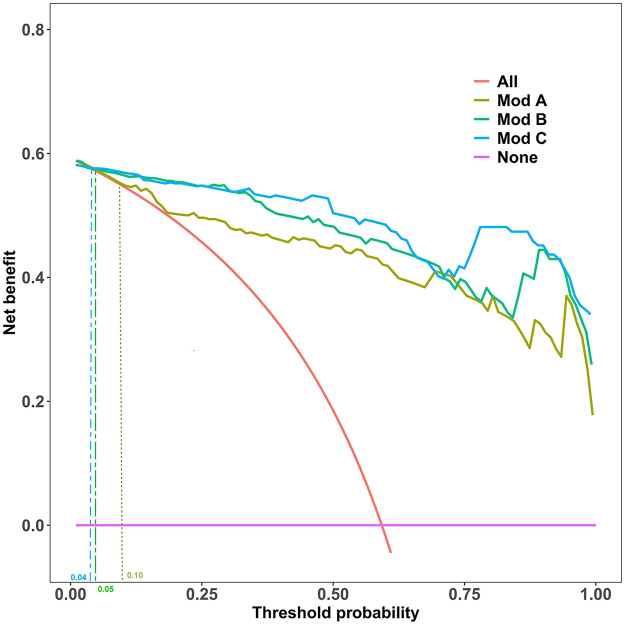
Decision Curve Analysis (DCA) for the Prediction Models. This analysis compares the net benefit of the three models against the “Treat All” and “Treat None” strategies across a range of threshold probabilities. Clinical utility, defined as when a model's curve surpasses the “Treat None” line, begins at threshold probabilities of 10% (Model A), 5% (Model B), and 4% (Model C).

Decision curve analysis (DCA), based on the original predicted probabilities, is central to evaluating the clinical value of a model. As shown in Figure 3, across a wide range of threshold probabilities, all three prediction models provided a superior net clinical benefit compared to the “treat all” or “treat none” strategies.

As summarized in Table 3, the clinical applicability of Model B and C was significantly better than Model A. Specifically, Model B provided net benefit from a very low threshold probability of 5% up to 100%, resulting in a useful threshold range length of 0.94. Model C had a slightly longer useful range (0.95), starting at a 4% threshold. Although Model C's range was marginally longer, its DCA curve highly overlapped with that of Model B across most of the range, visually reinforcing their highly similar clinical utility.

### 3.4 Nomogram for predicting persistent CAA risk based on final model B

**Figure 4:**
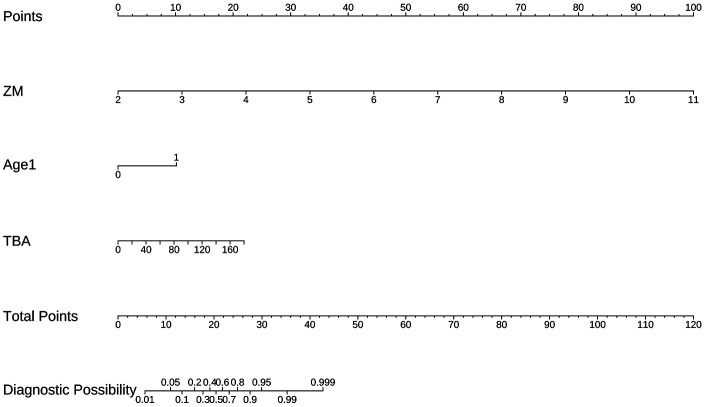
Nomogram for Predicting the Risk of Persistent CAA Based on Final Model B. The nomogram incorporates three predictors: coronary artery *Z*-score (ZM), age (Age1), and total bile acid (TBA). The individual score for each predictor is determined on the *Points* axis, and the sum of these scores is used to estimate the predicted probability of persistent coronary artery aneurysm on the *Total Points* axis.

Based on a comprehensive consideration of performance equivalence, model parsimony, and clinical utility, Model B was selected as the final prediction model. Performance Equivalence: Model B showed no statistically significant difference in discrimination from the more complex Model C (*p* = 0.389) and demonstrated good calibration. Maximum Parsimony: Model B achieved predictive performance equivalent to the 5-variable model using only 3 variables, potentially enhancing generalizability and clinical ease of use. Clinical Utility: Model B possessed a net clinical benefit nearly identical to Model C (useful range length 0.94 vs. 0.95) and could effectively guide clinical decisions starting from a 5% threshold probability. Based on the regression coefficients of Model B, a nomogram for predicting the risk of persistent CAA was constructed (Figure 4). This tool transforms the complex regression model into an easy-to-use visual scoring system. Clinicians can quickly determine the corresponding individualized risk probability on the chart based on a child's maximum coronary *Z*-score (ZM), age < 12 months (Age1), and total bile acid (TBA) level. The complete multivariable logistic regression coefficients, standard errors, odds ratios, and 95% confidence intervals for the final prediction model (Model B) are provided in Supplementary Table S1.

## 4 Discussion

This study focused on a clinically challenging population—children with Kawasaki disease (KD) who had already developed coronary artery aneurysms (CAAs) in the acute phase. Within this high-risk cohort, we innovatively applied bootstrap-LASSO stability selection to identify which children were likely to have persistent CAAs three months after onset. We successfully developed and validated a parsimonious prediction model (Model B) containing only three variables: maximum coronary *Z*-score (ZM), age <12 months (Age1), and total bile acid (TBA). This model not only accurately identified the risk of persistent CAA (corrected AUC: 0.933) but, more importantly, its exceptional clinical utility lies in its ability to further stratify children with acute-phase CAAs, precisely identifying the subgroup with potentially worse long-term outcomes, thereby providing crucial evidence for intensified follow-up and individualized therapy. Consequently, this predictive model amalgamates three key dimensions of pathophysiology: the anatomic dimension (ZM, quantifying initial injury magnitude), the host dimension (Age1, inferring regenerative capacity), and the immune-metabolic dimension (TBA, signaling persistent inflammatory dysregulation). This tripartite synergy offers a comprehensive framework for risk stratification, thereby marking a paradigm shift from reliance on purely anatomic criteria to a multifaceted prognostic approach.

### 4.1 From acute injury to long-term outcome: precision in risk stratification

This study sets the prediction “starting line” after acute-phase CAA has already occurred. At this specific stage, the focus of clinical decision-making shifts from “whether CAA will occur” to “whether this CAA injury will be persistent”. The maximum coronary *Z*-score (ZM), as the strongest predictor, directly reflects the severity of acute coronary damage and serves as an important indicator for predicting coronary lesion severity and regression potential, though its predictive power may be influenced by threshold selection, combined indicators, and population differences (15–17). This aligns with the pathophysiological consensus that more significant inflammatory damage to the vascular wall requires longer repair time and is more prone to leaving permanent structural changes (18–20). The inclusion of age <12 months (Age1) in the model suggests that being an infant is independently associated with a higher risk of persistent CAA., This aligns with emerging evidence suggesting that the pathophysiology of KD in infants may lead to more severe vascular injury or impaired repair mechanisms once coronary dilation occurs (21). By quantifying these factors, our study achieves precise risk re-stratification within the population of children with acute-phase CAAs. Furthermore, an important question regarding whether the extent of coronary involvement (i.e., multi-vessel disease) influences prognosis was addressed. We performed a supplementary analysis. Univariate analysis confirmed that the proportion of children with multi-vessel (≥2) involvement was significantly higher in the persistent CAA group than in the regressed group (53.8% vs. 20.0%, *p* = 0.003). However, when the binary variable “multi-vessel involvement (≥2 vessels)” was included in a multivariable logistic regression model alongside the core predictors (ZM, Age1, TBA), it failed to provide independent predictive information (adjusted odds ratio = 0.57, 95% CI: 0.10–3.37, *p* = 0.537). This finding carries a dual implication: clinically, it affirms that multi-vessel disease is a clear marker of greater disease severity; for predictive modeling, it indicates that the maximum *Z*-score (ZM), as a continuous variable, comprehensively captures the key anatomical risk information encompassing both the peak severity and extent of damage. Consequently, for predicting CAA persistence, the “peak intensity” of injury (ZM) constitutes a more robust and stable anatomical predictor than the mere binary classification of multi-vessel involvement. This further supports the selection of ZM as a core predictor.

### 4.2 TBA: A prognostic warning signal beyond liver function

In children with acute-phase CAAs, elevated total bile acid (TBA) emerged as an independent negative predictor, a finding of significant implications. In this context, TBA may be more than just a marker of liver involvement; its deeper significance potentially reflects the body's response state to severe systemic inflammation and associated metabolic disturbances (22). We hypothesize that during the acute phase of KD, severe systemic inflammation may lead to bile acid metabolism dysfunction and gut-liver axis disruption, resulting in elevated serum TBA levels. The signaling molecule function of elevated TBA might be far more important than its role as a liver injury marker.

Recent research has gradually revealed the crucial role of bile acids as signaling molecules in systemic inflammatory diseases. Their mechanism of action is particularly noteworthy in the context of KD, an acute vasculitis. Bile acids precisely regulate inflammatory signaling through the TGR5-cAMP-PKA axis. TGR5 activation induces PKA-mediated phosphorylation of NLRP3 (at Ser291), promoting its ubiquitination and degradation, thereby inhibiting inflammasome assembly (23). This anti-inflammatory effect is receptor-specific, as it is absent in TGR5-deficient monocytes (24). Substantial evidence confirms the NLRP3/IL-1β pathway as a major driver of KD pathophysiology, with mouse models and patient transcriptome data demonstrating NLRP3 involvement in the development of coronary arteritis (25–28). Similar to autoinflammatory diseases, KD patients exhibit ITPKC gene polymorphisms (affecting endoplasmic reticulum calcium homeostasis) and ER stress, which may activate NLRP3 through yet unidentified mechanisms (28, 29). Single-cell sequencing has revealed altered expression of bile acid metabolism (BAM)-related genes in specific immune cell subsets (e.g., CD8+ T cells) in KD patients, suggesting bile acid signaling may participate in immune regulation (30).

Therefore, measuring TBA in children with acute-phase CAA could help identify “concealed high-risk” individuals who exhibit significant systemic inflammatory responses and poor long-term coronary recovery potential, despite having only mild apparent liver dysfunction, going beyond traditional inflammatory markers like CRP. Consequently, elevated TBA levels in our study might reflect an imbalance between impaired TGR5-mediated anti-inflammatory pathways and excessive NLRP3 inflammasome activation. This imbalance could hinder timely coronary repair, ultimately leading to persistent aneurysms. This also provides new perspectives for future research exploring the modulation of bile acid metabolism or related signaling pathways to improve coronary outcomes in KD.

### 4.3 Clinical implications: guiding long-term management strategies for high-risk populations

The primary application of the developed nomogram is risk assessment of children with acute-phase CAA before discharge or shortly after the acute phase. By quickly calculating the risk probability, clinicians can objectively identify high-risk individuals, with particular emphasis oninfants (age <1 year), whom the model identifies as having a significantly elevated inherent risk for persistent injury. To facilitate precise calculation, a detailed score conversion table is provided in Supplementary Table S2, enabling risk estimation without relying solely on the graphical nomogram. For clinical integration, we propose a practical workflow: Risk Quantification: Use the nomogram or Supplementary Table S2 to calculate an individualized risk probability. Risk Stratification: Categorize patients into risk tiers (e.g., low, intermediate, high) based on this probability. The specific probability thresholds defining these tiers may be adapted based on local clinical judgment and resources. Management Triage: Match the risk tier to corresponding management intensity. This early identification allows for the timely enrollment of these children into stricter long-term management pathways, such as more frequent cardiac ultrasound follow-up, prolonged antiplatelet therapy, or even early consideration of secondary prevention medications (e.g., statins) to promote vascular repair (29). Decision curve analysis (DCA) provides objective evidence for this risk-based decision-making. Model B demonstrates net benefit starting from a very low threshold probability of 5%, indicating its clinical utility across a broad spectrum of decision thresholds. This supports the model's role in informing, rather than replacing, clinical judgment. In summary, this nomogram serves as a quantitative decision-support tool. It transforms clinical and laboratory data into an individualized risk estimate, providing an objective basis for tailoring follow-up strategy. Future work may focus on validating specific probability cut-offs for intervention in larger, prospective cohorts.

## 5 Limitations

This study has several limitations. First, as a retrospective study with data sourced from a single center, its results may be subject to selection bias, and future multicenter, prospective studies are needed for external validation to confirm its generalizability. Second, the study primarily focused on clinical variables and did not incorporate genetic or immunological biomarkers. Third, regarding interventions during follow-up, some children received rescue therapy (a second IVIG infusion combined with corticosteroids) for IVIG resistance. We did not include “corticosteroid use” as a separate predictor because it was an integral part of a standardized rescue protocol triggered solely by IVIG resistance. The variable “IVIG resistance” was itself included in our initial candidate pool and subjected to stability selection. Its relatively low selection frequency suggests that, in our cohort, the predictive information regarding the need for rescue therapy and its associated outcome was largely captured by the core variables in the final model (ZM, Age1, TBA). Therefore, our model estimates the “real-world” risk of persistent CAA within a standard clinical pathway that includes protocolized rescue therapy for IVIG-resistant cases. Nevertheless, the independent effect of rescue therapy on aneurysm regression cannot be fully isolated in this observational design. Future prospective studies could further elucidate its specific contribution under more controlled conditions. Fourth, elucidating the specific molecular mechanisms by which TBA influences coronary recovery is a crucial next step to connect this clinical finding with potential intervention targets. Finally, developing online calculators or mobile applications would be an important step in facilitating the clinical translation of this model.

## 6 Conclusion

In children with KD who have acute-phase CAAs, the prediction model and nomogram developed based on ZM, Age1, and TBA can effectively identify individuals at risk of persistent CAAs. This tool shows promise for optimizing the long-term management strategy for this high-risk group, enabling a shift from a “one-size-fits-all” follow-up approach to a risk-based precision medicine model, ultimately aiming to improve their long-term cardiovascular health.

## Data Availability

The raw data supporting the conclusions of this article will be made available by the authors, without undue reservation.
